# Potency of Fish Collagen as a Scaffold for Regenerative Medicine

**DOI:** 10.1155/2014/302932

**Published:** 2014-05-25

**Authors:** Shizuka Yamada, Kohei Yamamoto, Takeshi Ikeda, Kajiro Yanagiguchi, Yoshihiko Hayashi

**Affiliations:** Department of Cardiology, Nagasaki University Graduate School of Biomedical Sciences, Nagasaki 852-8588, Japan

## Abstract

Cells, growth factors, and scaffold are the crucial factors for tissue engineering. Recently, scaffolds consisting of natural polymers, such as collagen and gelatin, bioabsorbable synthetic polymers, such as polylactic acid and polyglycolic acid, and inorganic materials, such as hydroxyapatite, as well as composite materials have been rapidly developed. In particular, collagen is the most promising material for tissue engineering due to its biocompatibility and biodegradability. Collagen contains specific cell adhesion domains, including the arginine-glycine-aspartic acid (RGD) motif. After the integrin receptor on the cell surface binds to the RGD motif on the collagen molecule, cell adhesion is actively induced. This interaction contributes to the promotion of cell growth and differentiation and the regulation of various cell functions. However, it is difficult to use a pure collagen scaffold as a tissue engineering material due to its low mechanical strength. In order to make up for this disadvantage, collagen scaffolds are often modified using a cross-linker, such as gamma irradiation and carbodiimide. Taking into account the possibility of zoonosis, a variety of recent reports have been documented using fish collagen scaffolds. We herein review the potency of fish collagen scaffolds as well as associated problems to be addressed for use in regenerative medicine.

## 1. Natural Polymers as a Scaffold Material

Biomaterials (polymers) have been comprehensively reviewed by Silvestri et al. [1]. Collagen, gelatin, Matrigel, fibrin, alginate, cellulose, chitosan, hyaluronic acid, and silk fibroin have been investigated as bioactive polymers. Collagen is the major constituent of the extracellular matrix [2, 3]. Among natural polymers, bovine collagen, primarily that of type I, has long been used in biomedical applications as a hemostatic agent to treat tissue injuries [4]. After its regenerative properties were discovered, it was applied in 3D cultures for use in regenerative medicine [5]. As severe infections (zoonosis), including bovine spongiform encephalopathy, avian and swine influenza, and foot-and-mouth diseasein bovines, pigs, and buffalo, often occur worldwide, with respect to scaffold manufacturing, the use of bioactive natural organic materials originating from marine products is indispensable.

## 2. General Properties of Scaffold Materials for Use in Regenerative Medicine

The basic principle of tissue engineering is that cells, genes, and proteins are delivered via a degradable material, termed a scaffold, in order to regenerate tissue. This concept was first elucidated by Langer and colleagues [6–9]. These authors laid out the basic requirements for scaffolds as follows: (1) the material selected to support the matrix should be biocompatible and readily processed into the desired shape, (2) interactions between host cells and the material must be considered based on the structural and metabolic demands of the specific tissue, and (3) the performances of the matrix should be evaluated both* in vitro* and* in vivo* using quantitative molecular and histological assays. These principles constitute the foundation of tissue-engineering scaffold research and development.

A scaffold functions to (a) provide structural integrity and define the potential space for the engineered tissue, (b) guide the restructuring process involving the proliferation of donor cells and growth of the host tissue, (c) maintain a distance between parenchymal cells that permits the diffusion of gas and nutrients and possibly vasculature growth from the host bed, and (d) transmit tissue-specific mechanical forces to cue the behavior of cells within the material [10]. Based on these criteria, the sponge form is a suitable and reasonable scaffold structure [11].

Beyond identifying which factors affect tissue regeneration, it is difficult to determine which quantitative parameters can be used to characterize such regeneration-enhancing factors. Three scaffold-design parameters have been accepted to influence tissue regeneration: (i) the modification of the scaffold surface in order to enhance cell interactions, (ii) the controlled release of growth factors from the scaffold, and (iii) the use of scaffold mass transport [12].

Enhancing tissue regeneration by controlling cell-scaffold interactions and accommodating cellular metabolic demands based on the degree of scaffold diffusivity are two fundamental scaffold-design requirements outlined in the early 1990s [6, 9].

The concept of scaffold mass transport is characterized by scaffold diffusively and permeability. As with mechanical properties, native tissue diffusivity and permeability can be regarded as the starting point for defining scaffold-transport design targets [12]. One of the major goals of designed diffusivity and permeability is to control the rate of oxygen diffusion to cells in order to regenerate tissues. The partial oxygen pressure is a factor clearly affected by scaffold mass-transport characteristics, thus influencing cell differentiation. Most studies regarding the differentiation of progenitor cells and/or behavior of fully differentiated cells are based on required permeability and diffusivity values [13, 14].

## 3. Characteristics of Fish Collagen

### 3.1. Differences between Species

Fish type I collagen is unique in its extremely high solubility in dilute acid [15, 16] compared to avian and mammalian collagen. Compared with calf type I collagen, lower vertebrate type I collagen derived from bony fish and lamprey has been found to exhibit a high degree of structural similarity between species with respect to the *α*1 and *α*2 chains.

Collagen substrates are known to affect the growth characteristics of cells and modulate various aspects of cell behavior, such as cell adhesion, proliferation, and differentiation [17–26]. The disadvantages of collagen as a biomaterial for use in tissue repair include its low level of biomechanical stiffness and rapid rate of biodegradation [27].

Collagens are easily extracted and purified from wasted fish skins and bones with a high yield. Briefly, the cleaned skins, bones, and scales are generally extracted with acid solution with stirring. The extract is centrifuged by high speed refrigerated centrifuge, and then the supernatant is salted out by adding sodium chloride (NaCl). The resulting precipitates are collected by centrifugation. The precipitate is dissolved in acid solution and then any insoluble material is removed by centrifugation. The supernatant is salted out by adding NaCl. And the resulting precipitates are separated by centrifugation. The precipitate is dissolved in acid solution. After centrifugation, the supernatant is dialysed against dibasic sodium phosphate. The precipitate is obtained by centrifugation and then lyophilised. These procedures are performed at 4°C. Therefore, the use of fish collagen may contribute to the recycling of an unutilized resource, with consequent highly value-added production.

### 3.2. Chemical Properties

#### 3.2.1. Amino Acid Composition

The biochemical composition of fish collagen is thought to be different from that of mammalian collagen. For biochemical analyses, the application of strict conditions for sample preservation prior to collagen extraction is indispensable, as the stability of the hydroxyproline content strongly depends on the sampling procedure [28]. Several previous studies have demonstrated that the amino acid composition of fish collagen is similar to that of mammalian collagen [29–33] (Table 1). For example, glycine is the most abundant amino acid, accounting for more than 30% of all amino acids. In addition, the degree of hydroxylation of proline has been calculated to be 35–48%, similar to the level observed in mammalian tissues (approximately 45%) (Table 2). Furthermore, a linear relationship between the stability of collagen and the hydroxyproline content has generally been recognized.

#### 3.2.2. RGD Motif

The RGD motif is a representative amino acid sequence with cell adhesion properties that is comprised of arginine (Arg)-glycine (Gly)-aspartic acid (Asp). Arg-Gly-Asp-serine (Ser) sequences have been identified to constitute functional sites for fibronectin, the cell adhesion molecule [34]. Subsequently, it was proven that the cell adhesion properties of this motif do not change, even when Ser is substituted for valine, threonine, or alanine. Therefore, the RGD sequence is considered to be an intrinsic peptide with inherent cell adhesion properties. In addition to that observed in collagen, the RGD motif is located at functional sites in proteins, such as vitronectin, fibrinogen, laminin, tenascin, von Willebrand factor, and osteopontin. Cell adhesion molecules with RGD sequences are ligand proteins that contribute to adhesion between components of the extracellular matrix and communication among cells, with receptors composed of heterodimeric transmembrane proteins, called “integrins,” on the cell surface. Although the RGD motif is present in more than 100 types of proteins, the number of proteins able to function as cell adhesion molecules is limited, as mentioned above. Therefore, in addition to being exposed on the surface of proteins, the RGD domain exhibits a particular functional structure when the RGD motif binds to integrin, thus resulting in adhesion.

#### 3.2.3. Denaturation Temperature

Fish collagen fibrillar gels have not been studied, with the exception of shark collagen [35, 36], likely due to their low denaturation temperature (Td), which renders these materials difficult to handle. The Td of shark collagen solution is approximately 30°C [37], which results in the dissolution of the fibrillar gel of this collagen at 37°C [35]. Such features indicate that the gel cannot be used at the actual physical temperature required for human medical application. The Td of chum salmon is approximately 19°C [30, 38], which makes this material unstable at the physical temperature of the human body. As the denaturation temperature of fish collagen is lower than the mammalian body temperature, fish collagen melts when placed in contact with the human body for clinical application. Recently, collagen extracts derived from ray skin and the scales of tropical fish (tilapia), have been reported to have a Td of 33-34°C [33] and 35°C [39], respectively. Furthermore, improvements in collagen fibrillogenesis can be achieved with chemical cross-linking* in vitro*. This method brings the Td of salmon collagen to 55°C, and the biocompatible properties of this material have been demonstrated in several studies [32, 40]. Furthermore, it is very interesting that the degree of hydroxylation of proline of cold sea fish, for example, chum salmon, has been reported to be low (35–37%) [30, 38] compared to that of relatively warm sea fish (e.g., tilapia: 43%), a phenomenon related to the Td of the fish (Table 2).

#### 3.2.4. Cross-Linking for Stability

Numerous attempts have recently been made to use type I collagen as a biomaterial. The cross-linking methods employed to stabilize collagen can be divided into physical treatments, such as ultraviolet irradiation [41], gamma irradiation [42] and dehydrothermal treatment [41, 43–45], and chemical treatments, such as that involving glutaraldehyde [46], carbodiimide [45], or 1-ethyl-3-(3-dimethyl-aminopropyl)-carbodiimide (EDC) [47]. Chemical treatments confer remarkably high strength and stability to the collagen matrix, although they can result in potential cytotoxicity or poor biocompatibility [48], whereas physical treatments provide sufficient stability with no cytotoxicity [43, 49].

## 4. Biocompatibility

The primary reasons for using collagen include its excellent biocompatibility, low antigenicity [50], high level of direct cell adhesion [25], and high degree of biodegradability compared to chitin/chitosan and synthetic polymers [51]. The application of fish collagen as a scaffold for tissue engineering has been attempted [52, 53]. Our laboratory has also begun to evaluate the safety of fish (tilapia) collagen and have observed only very mild reactions in rat pulp induced by tilapia collagen, even at the initial stage of application (unpublished data).

Atelocollagen is a processed natural biomaterial produced from bovine type I collagen. It inherits useful biomaterial characteristics from collagen, including a low rate of inflammatory responses, high level of biocompatibility, and high degree of biodegradability [54, 55]. The components of collagen that are attributed to its immunogenicity, namely, telopeptides, are eliminated during atelocollagen production. Therefore, atelocollagen exhibits little immunogenicity [56]. The ability to obtain a substantial amount of collagen from fish waste (scales, skin, and bone) would result in the development of an alternative to bovine collagen for use in food, cosmetics, and biomedical materials.

Collagen scaffolds of jellyfish which is one of marine organisms as well as fishes display a highly porous and interconnected pore structure, which is useful for high-density cell seeding and provides an efficient nutrient and oxygen supply to the cells cultured in three-dimensional matrices. In order to determine whether jellyfish collagen evokes a specific inflammatory response compared to bovine collagen or gelatin, the levels of proinflammatory cytokines and antibodies were measured, and the changes in the population of immune cells following* in vivo* implantation were evaluated. Subsequently, jellyfish collagen was found to induce an immune response comparable to that stimulated by bovine collagen and/or gelatin [57].

Elastic salmon collagen (SC) vascular grafts have been prepared by incubating a mixture of acidic SC solution and fibrillogenesis-inducing buffer containing a cross-linking agent, water-soluble carbodiimide (WSC). Subsequently, re-cross-linking in ethanol solution containing WSC was performed. Upon subcutaneous placement in rat tissues, the SC grafts induced little inflammatory reactions [52].

Furthermore, collagen sponges with microporous structures derived from tilapia have been fabricated from reconstituted collagen fibrils using freeze drying and cross-linked via dehydrothermal (DHT) treatment or additional WSC treatment. Tests of pellet implantation into the paravertebral muscle in rabbits have demonstrated that tilapia collagen rarely induces inflammatory responses at one or four weeks after implantation, a finding that is statistically similar to that of porcine collagen and high-density polyethylene as a negative control [53].

## 5. Biodegradation

*In vitro* degradation studies (using collagenase solution) have demonstrated a higher level of stability among cross-linked scaffolds derived from tropical fresh water fish scale collagen, with only a ~50% reduction in mass after 30 days, whereas the uncrosslinked scaffold has been shown to degrade completely within four days. Furthermore, minimal immunological reactions were observed when the collagen solution was injected in mice treated with or without adjuvant therapy, without significant dilution of the sera [50]. These findings indicate that fish scale collagen is biocompatible in humans, with the potential to be used in tissue engineering applications.

Upon placement in subcutaneous tissues in rats, SC grafts gradually biodegrade. At one month after implantation, fibroblasts and macrophages begin to penetrate the surface of the graft, without signs of necrosis [52]. The rates of biodegradation of both collagen implants are similar, with the exception of DHT-treated tilapia collagen sponges at one week after implantation. In addition, various types of treated collagen have been reported to not disappear in tissue, even at four weeks after implantation [53].

## 6. Application for Use as a Biomaterial

### 6.1. Biomedical Applications

Numerous attempts have recently been made to use type I collagen as a biomaterial. Chemical treatments confer remarkably high strength and stability to the collagen matrix, although they can result in potential cytotoxicity or poor biocompatibility [48], whereas physical treatments provide sufficient stability with no cytotoxicity [43, 49].

Tissue engineering requires the application of a porous, biodegradable scaffold replicating the natural extracellular matrix, which serves to organize the cells spatially, providing them with environmental signals and direct site-specific cellular regulation [58]. The pore size, pore number, surface area, and pore wall morphology are widely recognized to be important parameters for scaffolds used in tissue engineering with respect to cell seeding, migration, growth, and new tissue formation [59, 60].

Polymer scaffolds are central to tissue engineering technology, as they direct a variety of cellular processes based on the structural and biochemical properties of the scaffold [59–61]. The materials used for scaffold fabrication not only determine the physical properties of biocompatibility, biodegradability, and mechanical stability, but also provide appropriate signals for directing the cellular processes that induce tissue formation [60, 62]. Collagen is an ideal scaffold or carrier for tissue engineering, as it supports many types of connective tissues, including skin, tendon, bone, cartilage, blood vessels, and ligaments [51, 63–73].

The application of jellyfish collagen containing telopeptides enhances the production of IgM in the human hybridoma cell line, HB4C5, as well as the production of IgM and IgG in human peripheral blood lymphocytes [74]. In addition, collagen derived from jellyfish stimulates both transcription and translation, thus enhancing immunoglobulin and cytokine production [75]. The* in vivo* responses of jellyfish atelocollagen have been investigated regarding safety for biological application in comparison with bovine collagen [57]. The resultant scaffold was found to exhibit a highly porous and interconnected pore structure, which is useful for high-density cell seeding, providing an efficient nutrient and an oxygen supply to the cells cultured in the three-dimensional matrix. In order to determine whether jellyfish atelocollagen evokes any specific inflammatory responses compared to that induced by bovine collagen or gelatin, this collagen was implanted onto the dorsal side in normal mice. The results demonstrated an immunological response comparable to that stimulated by bovine collagen and/or gelatin.

### 6.2. Dental Applications

**Figure 1:**
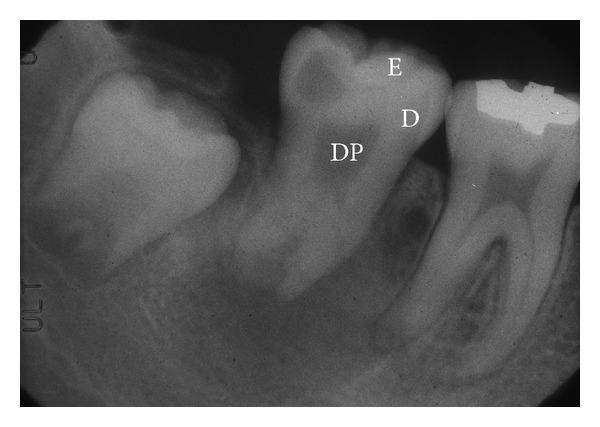
Structure of a tooth, DP: dental pulp, D: dentin, and E: enamel. The tooth has a unique structure, such that soft tissue called dental pulp is surrounded by hard tissue, including dentin and enamel.

The tooth has unique characteristics, such that soft and hard tissues exist together, with the hard tissue covering the soft tissue, the dental pulp (Figure 1).

#### 6.2.1. Soft Tissue

An interesting project regarding dental pulp regeneration is introduced and outlined in this paragraph. The Japanese Cabinet Office recently selected 24 projects to stimulate and promote Japanese medical innovation on November 18, 2008. In general, the projects were selected from among Highly Advanced Medical Treatment Development Fields in Japan. Only one project was selected in the field of dentistry: “The application of new treatments for dental caries/pulpitis through dentine/pulp regeneration using pulp stem cells” (representative of this special project: Dr. Misako Nakashima, Director, National Center for Geriatrics and Gerontology, Oobu City, Aichi, Japan). The basic principle of this research is to apply regenerative medicine using pulp stem cells, GCSF, and collagen solution to treat empty pulp extirpated cavities in patients with a history of pulpectomy and infected root canal treatment [76–86].

Although our department demonstrated that chitosan has numerous potential biological applications in the dental and medical fields [87–100], the disadvantage of chitosan is associated with severe inflammatory reactions, especially in the initial stage (1-2 weeks) after* in vivo* application [101]. The role of our department in the above-mentioned project is to evaluate the safety and stability of an alternative biomaterial, fish collagen. Furthermore, our goal is to establish standard operating procedures for the transportation of extracted tooth and isolated pulp stem cells in order to popularize this special treatment among general practitioners at dental clinics.

#### 6.2.2. Hard Tissue

The aim of hard tissue regenerative medicine in the field of dentistry is to treat defects of the alveolar and/or jaw bone originating from diverse etiologies. Several procedures have been developed to achieve periodontal regeneration, including bone graft placement, guided tissue/bone regeneration, and the use of various growth factors and/or host-modulating agents (e.g., Emdogain and parathyroid hormone) [102, 103].

The degradation or denaturation of salmon atelocollagen by *γ*-irradiation affects the rate of proliferation of MC3T3-E1 cells [42]. Human periodontal ligament fibroblasts are able to grow and exhibit a highly differentiated activity on salmon collagen gel as well as porcine collagen [40]. Including an appropriate functional scaffold (i.e., intricate 3D mesh composed of salmon collagen-coated fibers) may potentially improve the osteogenic potential of cultured periosteal sheets as a graft biomaterial both* in vitro* and* in vivo* [104]. Furthermore, fish collagen peptides promote posttranscriptional modification for collagen maturation and the gene expression for cell differentiation in osteoblastic cells [105, 106]. These findings indicate that fish collagen application* in situ* may promote hard tissue formation as both a scaffold for seeded cells and a nutritional factor for growth.

## 7. Problems regarding the Use of Fish Collagen in Regenerative Medicine

The safety of fish collagen has been investigated according to ISOstandards. It is important that further* in vitro* and* in vivo* studies be conducted in order to examine the immunological reactions induced by fish collagen prior to clinical application. In particular,* in situ* experiments using large animals, such as dogs, are indispensable, as inflammatory and immunological responses observed in animal experiments using rats and mice are generally thought to be weak compared to those observed in large animals.

## 8. Conclusions

Taking into account the possibility of zoonosis, a variety of recent reports using fish collagen as a scaffold have been published. Considering the factors involved in fabrication, such as the denaturation temperature and issues regarding biological safety, atelocollagen originating from tropical fish is thought to be a suitable biomaterial for use in clinical regenerative medicine. However, further animal experiments are needed before the material can be applied clinically.

## Figures and Tables

**Table 1:** Amino acid composition of bovine and tilapia.

Amino acids	Residues/1000	
	Bovine*	Tilapia**
Hydroxyproline	87	85.5
Asparaginic acid	35	44.0
Threonine	17	25.2
Serine	35	35.6
Glutamic acid	70	72.3
Proline	105	113.4
Glycine	296	332.3
Alanine	122	131.9
Valine	17	17.2
Methionine	17	9.6
Isoleucine	17	8.8
Leucine	35	22.4
Tyrosine	17	1.5
Phenylalanine	17	12.3
Histidine	7	5.6
Hydroxylysine	17	9
Lysine	35	23.6
Arginine	52	49.6

*Courtesy of Professor Mitsuo Yamauchi, North Carolina Oral Health Institute, NC, USA.

**Courtesy of Department of Protein Engineering, Nippi Inc., Tokyo, Japan.

**Table 2:** Degree of hydroxylation.

Fish	%
Squid	47.8
Carp	43.3
Eel	40.2
Common mackerel	41.1
Saury	40.5
Chum salmon	38.0
Tilapia	43.0
Tiger puffer	34.5
Dusky spinefoot	37.6
Sea chubs	40.4
Eagle ray	41.6
Red stingray	46.9
Yantai stingray	40.6

Control (bovine)	45.3
